# An ECG-Based Model for Left Ventricular Hypertrophy Detection: A Machine Learning Approach

**DOI:** 10.1109/OJEMB.2024.3509379

**Published:** 2024-11-29

**Authors:** Marion Taconné, Valentina D.A. Corino, Luca Mainardi

**Affiliations:** Department of Electronics, Information and Bioengineering (DEIB)Politecnico di Milano 18981 20133 Milano Italy; Department of Electronics, Information and Bioengineering (DEIB)Politecnico di Milano 18981 20133 Milano Italy; CardioTech LabIRCCS Centro Cardiologico Monzino 18607 20138 Milano Italy

**Keywords:** ECG features, left ventricular hypertrophy, ML classification

## Abstract

*Goal:* Despite the high incidence of left ventricular hypertrophy (LVH), clinical LVH-electrocardiography (ECG) criteria remain unsatisfactory due to low sensitivity. We propose an automatic LVH detection method based on ECG-extracted features and machine learning. *Methods:* ECG features were automatically extracted from two publicly available databases: PTB-XL with 2181 LVH and 9001 controls, and Georgia with 1012 LVH and 1387 controls. After preprocessing and feature extraction, the most relevant features from PTB-XL were selected to train three models: logistic regression, random forest (RF), and support vector machine (SVM). These classifiers, trained with selected features and a reduced set of five features, were evaluated on the Georgia database and compared with clinical LVH-ECG criteria. *Results:* RF and SVM models showed accuracies above 90% and increased sensitivity to above 86%, compared to clinical criteria achieving 38% at maximum. *Conclusions:* Automatic ECG-based LVH detection using machine learning outperforms conventional diagnostic criteria, benefiting clinical practice.

## I. Introduction

Left ventricular hypertrophy (LVH) is a strong predictor of cardiovascular morbidity and mortality, serving as a significant risk factor for cardiovascular disease [1]. Early detection of LVH can be beneficial for improving patient outcomes, enabling timely intervention and management of the condition. Although imaging techniques, particularly echocardiography, are considered the gold standard for LVH detection, they are not widely available, are expensive and time-consuming. In this context, electrocardiography (ECG) appears as an ideal modality for routine screening and monitoring of LVH.

Numerous LVH-ECG criteria, derived from visual inspection of the ECG recordings, have been proposed and adopted in clinical practice [2]. However, these criteria are often unsatisfactory due to their low accuracy [2], [3], [4]. While they are generally very specific (usually more than 80%), they lack the necessary sensitivity (often less than 50%) so that many LVH patients remain undetected and untreated.

In this context, integrating ECG-based features with advanced machine learning (ML) techniques may help in developing more accurate, automatic and reliable models for LVH detection. Several studies proposed their model to detect LVH from 12-lead ECG [5]: from simple decision tree [6] to more complex ML [7], [8], [9], [10], [11] and deep learning methods [12]. The key limitation of the above LVH detectors is their reliance on proprietary datasets [7], [10], [11] with a notable absence of external validation on publicly available datasets. External validation on large, open-source datasets is essential for objectively comparing the performance of LVH detectors, mitigating the potential biases inherent to proprietary datasets, and ensuring robustness across diverse clinical settings and recording types. Furthermore, when neither the database nor the trained models are made available, the potential for translation into clinical practice is severely limited.

In this paper, we addressed the aforementioned limitations by developing a novel LVH classifier, trained and validated on open-source datasets. To enhance transparency in the decision-making process, we disclosed the selected features and verified their significance through SHAP explainability analysis. Furthermore, to facilitate translation into clinical practice, we trained a simplified version of our classifier using only the five most relevant features.

The paper is organized as follows: Section II outlines the database, the proposed ML method, as well as the state-of-the-art clinical criteria; Section III presents the feature selection, training, and testing results, followed by the external validation results and the comparison with the clinical criteria; Section IV discusses the findings; and Section V concludes the paper.

## II. Material and Methods

### A. Database

Two publicly available datasets were analyzed in this study: The PTB-XL ECG database from Physikalisch-Technische Bundesanstalt [13] and the Georgia 12-lead ECG Challenge Database [14]. The PTB-XL ECG database was used for model building and contains 10-second, 500Hz sampled, 12-lead ECGs from 18,885 patients. ECGs labeled ‘NORM' and ‘LVH’ were extracted, excluding poor-quality recordings. The quality control was provided by the ECGDeli package [15] during the ECG template creation, when the signals without a minimum of beats with low deviation are excluded. After processing, a total of 2181 LVH and 9001 control patients' ECGs were included.

For external validation, we used the Georgia 12-lead ECG Challenge Database from Emory University, USA. This database also contains 10-second, 500Hz sampled ECGs. Applying the same selection criteria, we included 1012 LVH patients and 1387 controls ECGs for the testing phase.

Both databases are available in the Physionet challenge database repository [14].

### B. ECG Features Extraction

To perform classification, several representative features were extracted from the 12-lead ECG signal. They can be divided in three classes:

#### 1) Morphological Features

**Fig. 1.**
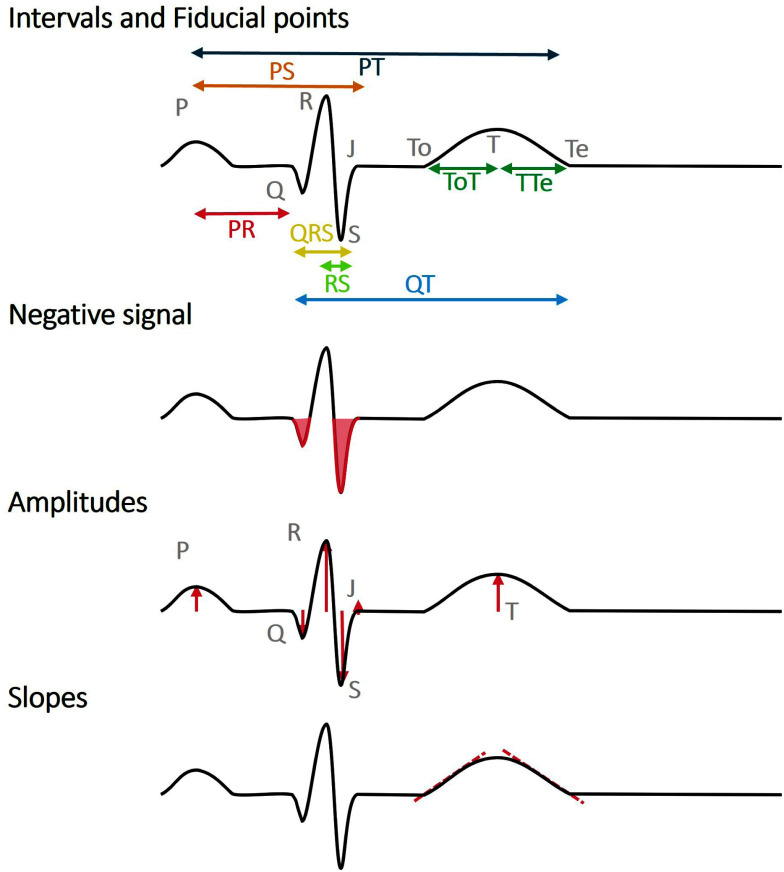
Morphological features extraction: Interval width, negative % of the signal, amplitude of the fiducial points and slopes of the waves.

A total of 19 features were automatically computed in each of the 12 leads after a step of fiducial points localization, automatically performed using the open-source ECGDeli package [15] executed in MATLAB R2023b. These include measures of wave amplitude and duration, traditionally extracted from standard ECG leads (Fig. 1), namely:

• Fiducial point amplitude: P, Q, R, S, J, T.
• Ratio of median fiducial amplitudes: R/P and R/T.
• Interval length: PR, PS, PT, QT, QRS, RS, T offset (To)T, TTend (Te).
• Ascending and descending slope of the T wave.
• Negative percentage of QRS.

Waves' amplitudes were extracted on each beat, and their median value was retained for successive analysis.

#### 2) Decomposition-Derived Features

After creating an average QRS complex for each lead, the average complexes were decomposed using the Hermite transform [16], which has been proved to provide a compact descriptor of QRS morphology. Briefly, each QRS complex ($s$) is approximated by a linear combination ($q$) of Hermite functions ($\Phi _{i}$):

\begin{equation*}
q(t) = \sum _{i=0}^{N_{q}-1}a_{i}\Phi _{i}(t) \tag{1}
\end{equation*}

where $a_{i}$ are the coefficients and $N_{q}$ is the number of Hermite functions used. In the study, we set $N_{q}=4$. This Hermite decomposition was applied separately to the average QRS of each lead, resulting in a total of 48 coefficients, e.g., $4 \times 12 \ (N_{q} \times \text{Leads}$), for each patient. Additionally, the root-mean-square error (RMSE) between the approximated ($q(t)$) and original QRS signals ($s(t)$) was computed:

\begin{equation*}
RMSE(s, q) = \sqrt{ \frac{1}{N_{s}}\sum _{i=0}^{N_{s}-1}(s(t_{i}) - q(t_{i}))^{2}} \tag{2}
\end{equation*}

where $N_{s}$ is the number of samples of the averaged QRS complex.

#### 3) Model-Derived Features

The V-index estimates spatial heterogeneity of ventricular myocytes' repolarization times ($\sigma _{v}$) [17], intended as the standard deviation of the repolarization times across the ventricles [18].

\begin{equation*}
V_{index} = \frac{std[w_{2}(i)]}{std[w_{2}(i)]} \approx \sigma _{v} \tag{3}
\end{equation*}

where $w_{1}$ and $w_{2}$ are the coefficients of the second-order approximation of the T-wave ($\boldsymbol{\Psi }$):

\begin{equation*}
\boldsymbol{\Psi } \approx w_{1} \bm {T}_{\boldsymbol{d}} + w_{2} \dot{\boldsymbol{T}_{d}} \tag{4}
\end{equation*}

$\bm {T}_{\boldsymbol{d}}$ is the vector of the dominant T-wave [19].

### C. ML Method

**Fig. 2.**
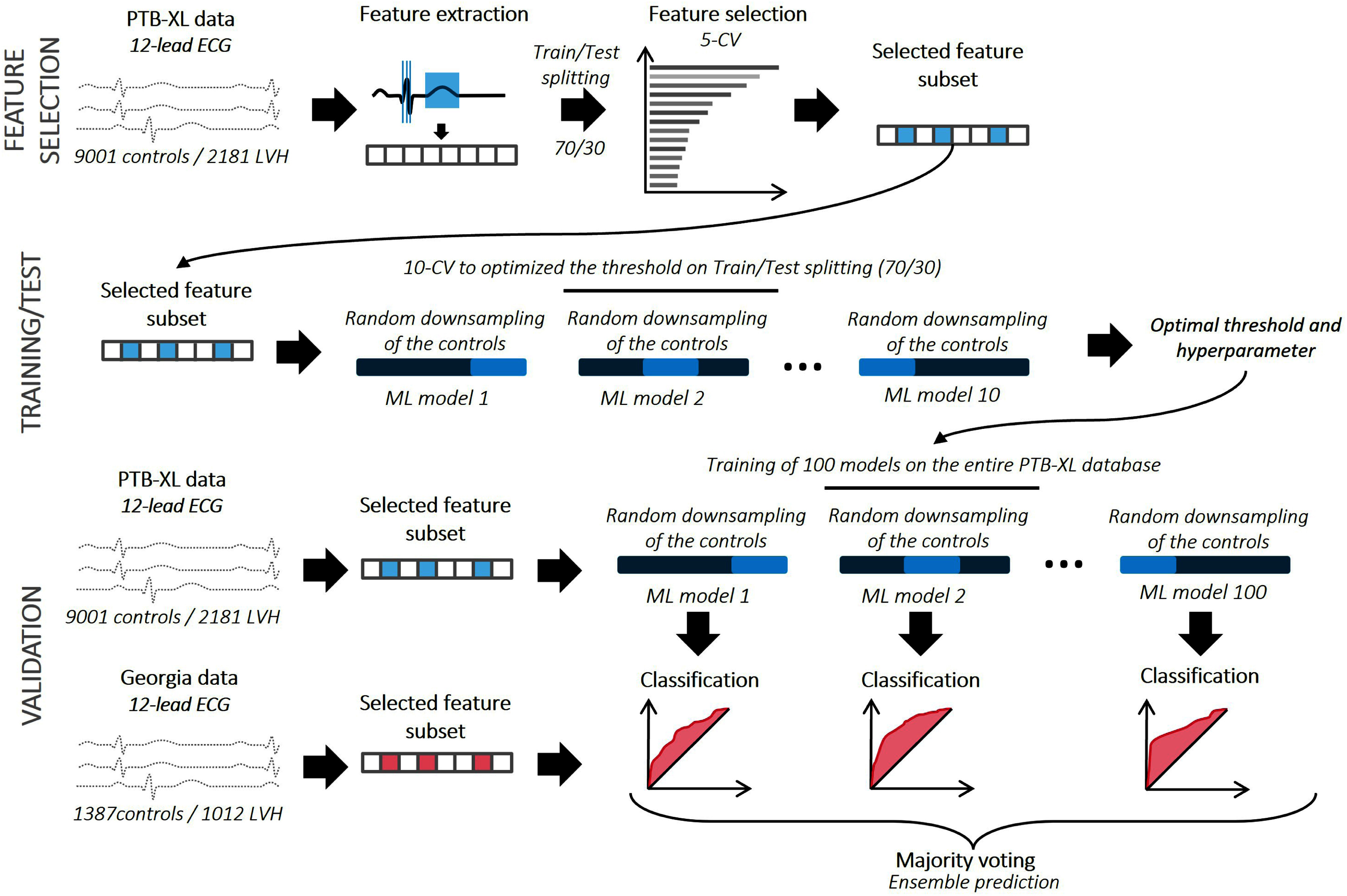
Methodological steps separated in training-test (top) and validation phase (bottom). The hundreds of training models are gathered to form a final ensemble model.

LVH classification was performed using three supervised ML algorithms: logistic regression (LogReg), random forest (RF) [20] and support vector machine (SVM) [21] such in [22]. A three steps procedure was designed, where we firstly selected the best feature set, then optimized the threshold and parameters, and finally validated the model on the external database. Fig. 2 sums up the three–steps procedures applied to train each model.

#### 1) Feature Selection Process

The objective of this step is to identify the most relevant features for LVH classification, simplifying models, reducing computation time, and minimizing overfitting.

Firstly, the PTB-XL database was split into a training (70%) and testing (30%) sets. Feature selection was performed on the training set using the sequential floating forward selection (SFFS) method in Scikit-Learn [23], [24]. SFFS is an iterative algorithm that dynamically adds and removes features to find the best combination, balancing model performance and feature complexity. At each step, the algorithm evaluates which feature improves the model the most, based on the area under the curve (AUC), and adds it. The process also allows backward elimination, where previously selected features can be removed if they no longer contribute significantly. To reduce bias from a specific train/test split, the procedure was repeated five times using Monte Carlo cross-validation. This identified the optimal feature combination for each ML model, resulting in distinct feature subsets. Additionally, the first five features selected by the SFFS algorithm were also used to create simpler models and evaluate the impact of number of features on performance.

#### 2) Model Training and Testing

Using its specific feature set, each ML model was trained on the PTB-XL database using 10-fold cross-validation to classify LVH patients versus normal individuals. Given the class imbalance, where LVH cases are the minority, the training set of each fold was balanced by down-sampling the majority class (i.e., the normal cases). This balancing procedure was independently performed for each fold.

The optimal classification threshold, $\hat{\theta }$, was identified as the point on the ROC curve that minimizes the distance to the point $P(0,1)$, namely:

\begin{equation*}
\hat{\theta } = \min _{\theta } \lbrace \text{FPR}\left(\theta \right)^{2} + (1-\text{TPR}\left(\theta \right))^{2} \rbrace \tag{5}
\end{equation*}

where TPR and FPR are the true and false positive rates.

#### 3) External Validation

After having been trained on the PTB-XL database, the three models were validated on the Georgia ECG Challenge Database. To emphasize the potential impact of majority label downsampling, 100 repetitions were conducted. The models' performance was evaluated based on sensitivity, specificity, accuracy, and AUC. Finally, a voting classifier was constructed from these 100 models to propose a unique classifier. The voting classifier predicts the class label based on the majority rule, where each of the 100 classifiers votes for a predicted label, and the majority label is proposed as the final predicted label (in case of a tie, the first model's prediction is taken).

#### 4) Model Explainability

In the pursuit of explainable AI and to link the ECG-extracted criteria automatically selected to physiological knowledge, the SHAP (SHapley Additive exPlanations) analysis was applied [25]. This analysis assigns an importance value to each feature for a particular prediction. SHAP can be applied to any model prediction and guarantees a unique solution, it was applied to the three final models.

### D. Clinical Criteria

To compare the proposed methods with clinical state–of–the–art, we computed a series of clinically adopted LVH criteria report in [2]. We included the criteria requiring the ECG signal only, excluding those needing sex, age, or specific pathological conditions. A total of 22 clinical criteria were calculated. They are listed in Table I.

**TABLE I:** Selected Subset of the LVH Criteria Based on [2]

**Criteria**	**Paper**	**Study Year**	**Abbreviation**
$ (R\, (I) - S\, (I)) + (S\, (III) - R\, (III)) > 16mm$	[26]	1914	Lewis
$ R\, (I) - S\, (III) > 25mm$	[27]	1943	Gubner1
$ R\, (I) > 15mm$	[27]	1943	Gubner2
$ R\, (aVL) > 11mm$	[28]	1949	Sokolow-Lyon1
$ R\, (aVF) > 20mm$	[29]	1949	Goldberger
$ Q\, or\, S\, (aVR) > 19mm$	[30]	1950	Schack
$ R\, +S\, (any\, limb\, lead) > 19mm$	[31]	1968	Romhilt1
$ S\, (V1) > 23mm$	[32]	1944	Wilson1
$ S\, (V2) > 25mm$	[33]	1964	Mazzoleni
$ S\, (V1) + R\, (V5) > 35mm$	[28]	1949	Sokolow-Lyon2
$ S\, (V2) + R\, (V5\, or\, V6) > 45mm$	[34]	1969	Romhilt2
$ S\, (V1\, or V2) + R\, (V5\, or\, V6) > 35mm$	[35]	1984	Murphy
$ S\, (V1\, or V2) + R\, (V6) > 40mm$	[36]	1957	Grant1
$ R + S\, (any\, precordial\, lead) > 35mm$	[36]	1957	Grant2
$R\, (V5) : R\, (V6) > 1.0$	[37]	1962	Holt
$ R (any\, precordial\, lead) > 26mm$	[38]	1958	McPhie
$ S\, (V2) + R\, (V4\, or\, V5) > 45mm$	[39]	1956	Wolff
$ R\, (V5) > 33mm$	[32]	1944	Wilson2
$ R\, (V6) > 25mm$	[32]	1944	Wilson3
$ Total\, 12-lead\, voltage > 175mm$	[40]	1982	Siegel
$ (R\, (aVL) + S\, (V3)) \cdot \, \text{QRS duration} > 2436mm/sec$	[41]	1992	Molloy1
$ Total\, 12-lead\, voltage \cdot \, \text{QRS duration} > 2436mm/sec$	[41]	1992	Molloy2

These scores were computed on the training/test database using automatically extracted fiducial point amplitudes and intervals. The five criteria, which had the best AUC on the taining/test dataset, were also computed on the validation database, compared with the ML methods, and plotted alongside the models' ROC curves. Additionally, these five criteria were combined (at least one of the five) for evaluation on the validation database.

## III. Results

### A. Hermite Approximation

A Hermite approximation of the QRS complex of each lead was computed for each patient. The fitting quality is quantified by the RMSE score. The average RMSE on the 12 leads was comparable on the two databases, being 38$\pm 42\,\mu$V for the PTB-XL database and 39$\pm 30\,\mu$V for the Georgia database. Examples of Hermite approximations are provided in the Supplementary Materials. Hermite functions well fit QRS complex signals without fragmentation, resulting in lower fitting errors for simpler QRS morphologies.

### B. Feature Selection Process

**Fig. 3.**
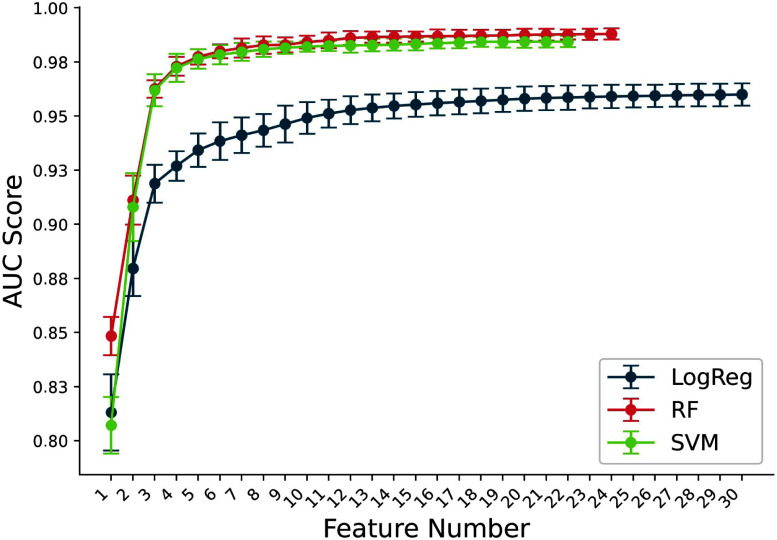
Mean area under the curve (AUC) during feature selection process, reorder by number of features. The three ML models are presented: logistic regression (LogReg), random forest (RF) and support vector machine (SVM).

Fig. 3 and Table II represent the SFFS process and results. Feature selection stopped at 22 and 24 features for the RF and SVM models, respectively, as no further AUC increase was observed up to 30 features.

**TABLE II:** Features Selected by the SFFS for the 3 Models: Logistic Regression (LogReg), Random Forest (RF) and Support Vector Machine (SVM)

**N**	**LogReg**	**RF**	**SVM**
1	T slope des (aVR)	R/T ratio (V6)	T slope des (aVR)
2	R amplitude (I)	R amplitude (V5)	R amplitude (V5)
3	R amplitude (V6)	R amplitude (III)	R amplitude (aVL)
4	Herm RMSE (V5)	R amplitude (V1)	R amplitude (V1)
5	R amplitude (V1)	T slope des (aVR)	P amplitude (aVR)
6	PS interval (II)	PS interval (II)	1st Herm coeff (I)
7	Herm RMSE (aVF)	Herm RMSE (I)	T amplitude (III)
8	R amplitude (V3)	R amplitude (V2)	T des slope (V6)
9	R amplitude (V5)	Herm RMSE (V6)	1st Herm coef (V2)
10	1st Herm coeff (III)	R amplitude (I)	T slope asc (aVR)
11	RS interval (V5)	P amplitude (I)	2nd Herm coef (V2)
12	Herm RMSE (V6)	R amplitude (II)	R ampltude (V6)
13	Q amplitude (V5)	V index	T slope des (V5)
14	Herm RMSE (III)	QT interval (V4)	R amplitude (III)
15	QRS % neg (V5)	P amplitude (III)	PS interval (aVF)
16	PT interval (V2)	S amplitude (III)	4th Herm coef (V4)
17	P amplitude (I)	RS interval (aVR)	J amplitude (V5)
18	Herm RMSE (aVL)	Q amplitude (avF)	S amplitude (V6)
19	P amplitude (V1)	3rd Herm coef (aVF)	R amplitude (aVR)
20	RS interval (V4)	PR interval (V4)	J amplitude (V4)
21	TTe interval (I)	ToT interval (V6)	T slope des (III)
22	Herm RMSE (V1)	S amplitude (I)	R/T ratio (V3)
23	QT interval (V4)	4th Herm coef (V5)	
24	Herm RMSE (II)	R/T (V4)	
25	J amplitude (aVR)		
26	S amplitude (I)		
27	Herm RMSE (avR)		
28	Q amplitude (V6)		
29	J amplitude (II)		
30	J amplitude (V3)		

The SFFS algorithms consistently selected similar features across the ML methods. Among the first five features, $R \ amplitude \ (V1)$ and $T \ slope \ des \ (aVR)$ were present across all three algorithms.

Notably, all leads are represented in the selected features.

### C. Model Training and Testing

The first selected features by ML (Table II) exhibit a clear association with clinical criteria found in the literature (Table I). For instance, the $R$ amplitude frequently appears in the feature selection. It is among the top five features for lead $V1$ across all three ML methods and appears in leads $I$, $aVL$, $V5$, and $V6$. These features can be linked with the [26], [27], [31] criteria for lead I, [28] for lead $aVL$, [38] for all the precordial lead, and various criteria for leads V5 and V6 such as [28], [32], [34], [35], [36], [37]. In all these cases, the threshold amplitude changes and can be combined with other measurements. This is similar to how machine learning, particularly decision trees, links features together step by step. The $S$ amplitude appears less frequently, represented only once in each ML model. However, $T$ and $P$ waves also show their significance in the feature selection process.

The training process optimized the number of random forest trees at 150. Additionally, the models' cutoff values were determined based on results from the PTB-XL database. The AUC values on the test set are: 0.953$\pm$0.004 and 0.929$\pm$0.004 for the LogReg with 30 and 5 features, 0.981$\pm$0.002 and 0.971$\pm$0.002 for the RF with 24 and 5 features, 0.979$\pm$0.002 and 0.971$\pm$0.002 for the SVM with 22 features and 5 features.

### D. External Validation

After training on the PTB-XL database, the model was applied to the Georgia database, with validation repeated 100 times to ensure that random downsampling had no major effect.

The AUC values from training to validation changed from 0.953$\pm$0.004 to 0.926$\pm$0.001 for logistic regression, from 0.981$\pm$0.002 to 0.976$\pm$0.001 for the RF with 24 features, and from 0.979$\pm$0.002 to 0.978$\pm$0.001 for the SVM with 22 features (all 3 statistically significant p-value$< $0.001).

**Fig. 4.**
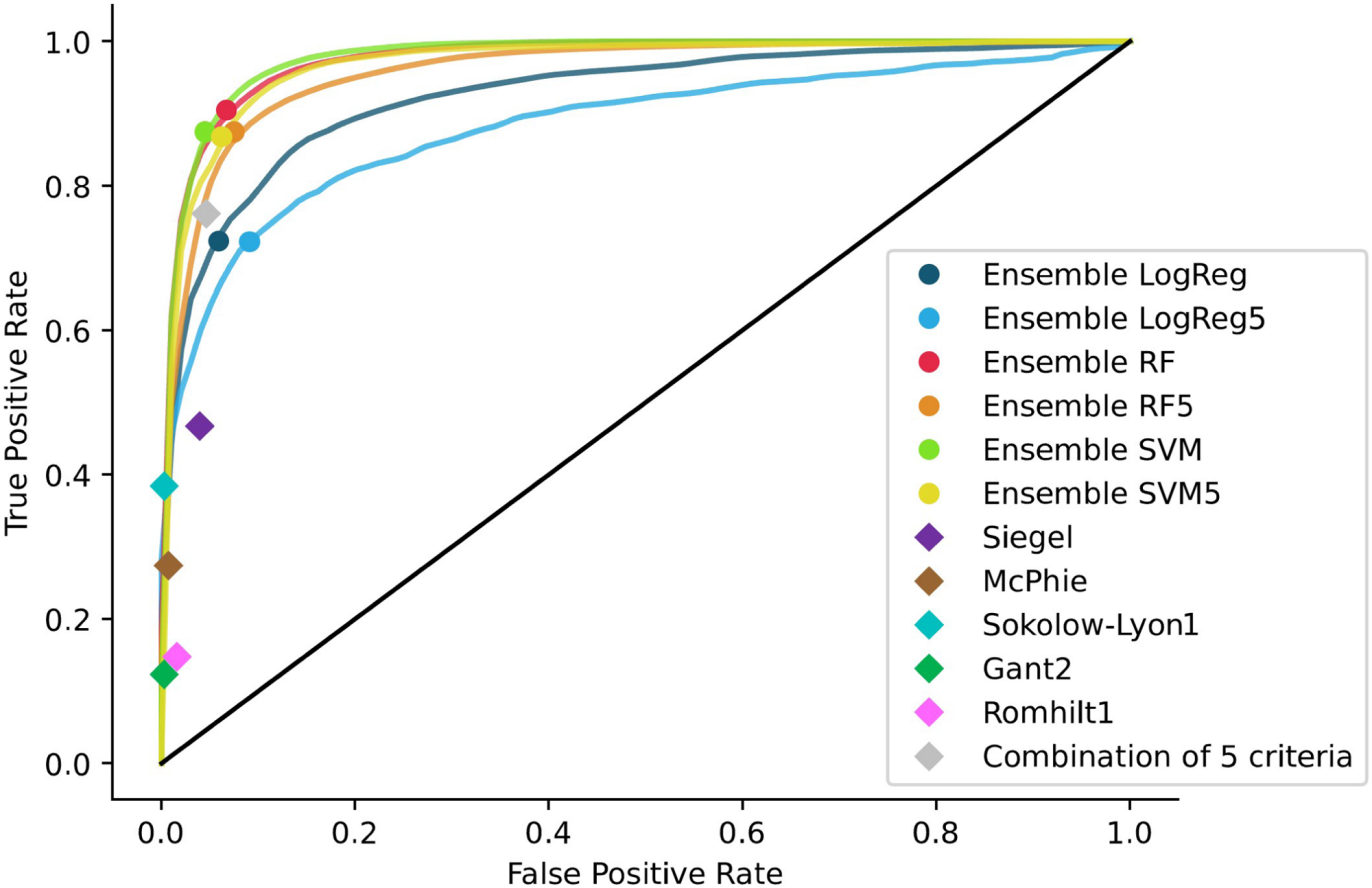
Mean ROC curves of the 3 algorithms on the external validation database: Logistic Regression (LogReg), Random Forest (RF) and Support Vector Machine (SVM) for the selected features up to 30 features and to the reduced to 5 features: LogReg5, RF5, SVM5, with the point of their equivalent ensemble method. The best 5 clinical criteria: Siegel [40], Mc Phie [38], Sokolow-Lyon [28], Gant2 [36], Romhilt1 [31], and their combination are also plotted, on the external validation database.

Fig. 4 shows the ROC curves for the three algorithms with feature sets of 5 and up to 30 features. The SVM with 22 features exhibited the best AUC result at 0.978$\pm$0.001, followed by the RF with 24 features at 0.976$\pm$0.001. Simpler models with five features, like SVM5 and RF5, also performed well with AUCs of 0.969$\pm$0.001 and 0.959$\pm$0.002, respectively.

The consistent standard deviation values across models indicate that downsampling of the control group did not significantly affect model performance.

**Fig. 5.**
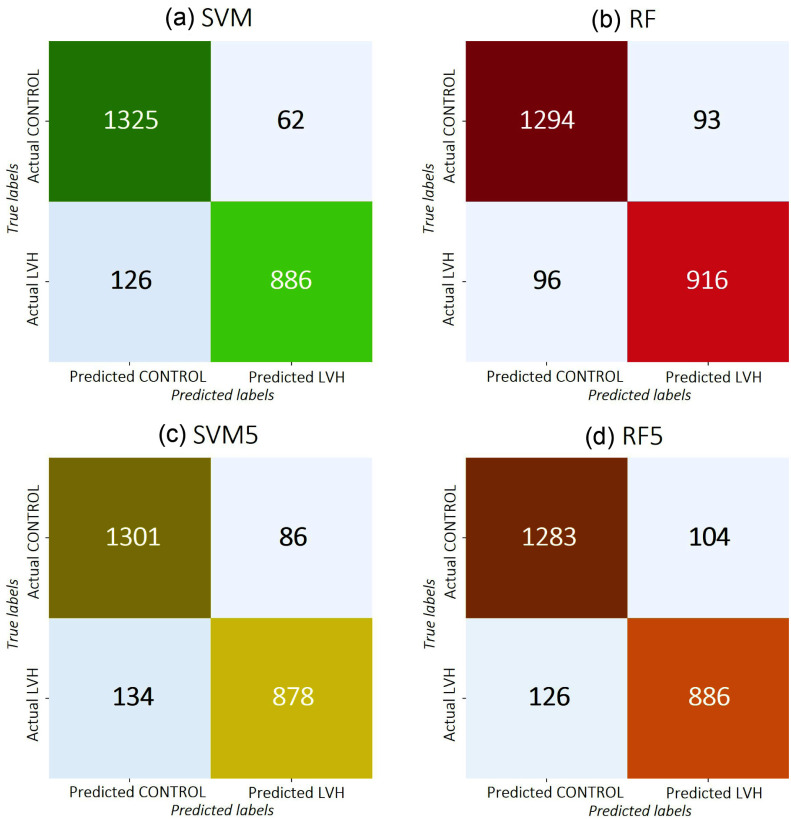
Confusion matrix of the ensemble models: (a) SVM, (b) RF, (c) SVM5, and (d) RF5 on the external validation database.

The ensemble methods reflect the individual models they are composed of, with similar fitting results. Table III summarizes the mean AUC, sensitivity, specificity, and accuracy of the six ensemble models. SVM and RF, as well as SVM5 and RF5, exhibit almost identical results, with slightly better performance for the SVM variants. Two other ML classifiers are also provided in Supplementary Materials. Fig. 5 displays their confusion matrices, which are well-balanced.

**TABLE III:** Sensitivity, Specificity, and Accuracy of the Ensemble Method for the 6 Types of Model on the Validation Database: Logistic Regression (LogReg), Random Forest (RF) and Support Vector Machine (SVM) for the Selected Features up to 30 Features and to the Reduced to 5 Features

	Sensitivity	Specificity	Accuracy	Balanced accuracy
Ensemble LogReg	0.724	0.941	0.850	0.833
Ensemble LogReg5	0.723	0.909	0.831	0.816
Ensemble RF	0.905	0.933	0.921	0.919
Ensemble RF5	0.875	0.925	0.904	0.900
Ensemble SVM	0.875	0.955	0.922	0.915
Ensemble SVM5	0.868	0.938	0.908	0.903
Siegel	0.467	0.960	0.752	0.714
McPhie	0.275	0.993	0.690	0.634
Sokolow-Lyon1	0.384	0.997	0.739	0.691
Gant2	0.124	0.997	0.629	0.561
Romhilt1	0.148	0.984	0.632	0.566
Combination of 5 clinical criteria	0.762	0.954	0.873	0.858

The Sensitivity, Specificity, and Accuracy of the 5 Best literature/clinical Criteria Were Also Added

### E. Clinical Criteria Comparison

The top five criteria (based on AUC on the PTB-XL database) were $Siegel$, $McPhie$, $Sokolow-Lyon1$, $Grant2$, and $Romhilt1$. These were computed on the validation database, along with their combination. Their plots are shown with the mean ML model ROC curves in Fig. 4, and their sensitivity, specificity, and accuracies are summarized in Table III. All individual criteria exhibit high specificity (over 0.96) but very poor sensitivity (below 0.47). However, combining these five criteria maintains high specificity while significantly increasing sensitivity to 0.762. This combination, easily applicable in clinical practice, outperforms existing criteria.

### F. Model Explainability

The SHAP results for the three models are detailed in the Supplementary Materials. The feature importance aligns closely with the feature selection order, with $R$ amplitude consistently ranked high. These results help understand each feature's impact on the validation database and its influence on the classification outcome. For instance, high $R$ amplitude in $V5$ or $V6$ positively influences the model, while deeper $R$ amplitudes in $V1$ negatively impact it. Similarly, a low $T \ slope \ des$ in $aVR$ is concordant with the fact that on this lead, healthy patients have a higher and positive slope. On the other hand, on $V5$ or $V6$, LVH patients present deeper T waves that imply high $T \ slope \ des$.

Regarding Hermite features, high RMSE values positively impact the model, indicating more complex signal morphologies. This suggests that $Herm \ RMSE$ reflects both signal quality and complexity. Additionally, lower $1st \ Herm \ coef$ values seem to correlate more strongly with LVH. In fact, bigger $1st \ Herm \ coef$ denote smoother shapes that seem coherent with control healthy patients.

## IV. Discussion

This study developed LVH classifiers based on automatically extracted ECG features, comparing them with established clinical criteria. Using publicly available 12-lead ECG databases, the SVM and RF models emerged as the top models. The models were trained on automatically extracted features, but these features could also be manually extracted from ECG recordings, including paper ECG. Using only five features, they showed that even simplified models yielded promising results suitable for clinical use.

### A. Feature Extraction and Selection

Our study utilized both classical and more complex features extracted from 12-lead ECGs. The large number of extracted features aimed to provide a comprehensive view of electrical activity from multiple angles and throughout the entire cardiac cycle. Fiducial points were extracted, and amplitudes and intervals were measured from all lead signals. Additional features such as T wave slopes and percentage of negative signal were also incorporated. Moreover, to capture features representing QRS morphology, Hermite approximation coefficients and RMSE were computed.

The feature selection process is crucial in every ML method, as it helps select meaningful features relevant to the objective while avoiding overfitting. In this study, the selected features included a mix of limb and precordial leads. The most relevant features in LVH classification were primarily related to the QRS complex, with R amplitude being prominently represented. This aligns with existing literature that has proposed numerous cutoff criteria based on R measurements. However, some features appeared without link to international guideline recommendations, such as the slope of the second part of the T wave. However, other studies [6], [7], [12] also identified the T wave amplitude, reflecting abnormal repolarization, as an indicator of LVH.

### B. Literature/Clinical Criteria

Current ECG criteria for classifying LVH exhibit very high specificity but lack satisfactory sensitivity. By combining the best literature criteria, we were able to increase sensitivity significantly without compromising specificity. Unlike other studies, where individual literature criteria were used, we combined them to mimic clinical practice, where multiple criteria are often considered diagnosing LVH.

Concerning the similar studies on ML detection of LVH (regrouped in the Supplementary Materials), those reporting better sensitivity, such as [10], [11], may have achieved impressive results due to their selected military population, rather than the absence of external validation. In contrast, a comparable study with external validation [12] demonstrated lower sensitivity (0.496) and similar specificity (0.936) using a deep learning method. Another study [6] managed to raise sensitivity to 0.743, but this came at the expense of specificity, which dropped to 0.687.

### C. Provided Models

ML models provided in this study offer the flexibility to adjust thresholds to meet specific sensitivity or specificity requirements. As mentioned, the training and validation were done on open access databases, that allowed retraining or modification if needed. Moreover, the train models are provided as well as the extracted features so they could be directly used on new ECG measurements.

### D. Limitations and Perspectives

A key limitation is the lack of detail on the labeling process of the databases [13], [14]. In fact, the diagnostic methodology is not thoroughly detailed and does not appear to have been reviewed by a second independent cardiologist. In our case, additional examinations, such as echocardiography and MRI could be highly valuable for quantifying hypertrophy and providing a more precise analysis of its localization.

Future work should consider multi-class classification, including right ventricular hypertrophy and valvular overload. More complex classification methods and integrating multiple diagnosis labels should also be explored.

Since this classification requires only a few parameters and the feature detection is fully automatic, a future step could involve integrating this classifier into ECG software, providing a LVH pre–diagnosis immediately after the ECG measurement. A robust LVH detection would enable real-time clinical decision support, streamlining the diagnostic process while also serving as an effective screening tool to reduce the need for unnecessary imaging diagnostics.

## V. Conclusion

Machine learning techniques significantly enhance LVH detection when integrated with ECG-derived features. The SVM and RF classifiers, achieving accuracy rates above 90% and sensitivity rates exceeding 86%, outperformed conventional criteria. The ability to tune ML models provides a distinct advantage, and successful validation on an independent open-source database underscores the robustness and generalizability of our approach. This study introduces an accessible and scalable approach that could be integrated into clinical practice, offering improved, real-time LVH detection and personalized patient care.

## Supplementary Materials

Additional figures and detailed information are provided in Supplementary Materials.

Supplementary Materials

## Author contributions

M.T., V.C., L.M. formed the concept and methodology of the study. M.T. conducted the investigation. V.C. and L.M. supervised the project and acquired funding. M.T. wrote the initial manuscript draft and created data visualizations with input from all authors. All authors contributed to and approved the final manuscript.

## Conflict of Interest

The authors declare that they have no competing interests.

## References

[1] M. R. Movahed, R. Ramaraj, C. Manrique, and M. Hashemzadeh, “Left ventricular hypertrophy is independently associated with all-cause mortality,” Amer. J. Cardiovasc. Dis., vol. 12, no. 1, pp. 38–41, 2022. 35291507 PMC8918741

[2] E. W. Hancock, B. J. Deal, D. M. Mirvis, P. Okin, P. Kligfield, and L. S. Gettes, “AHA/ACCF/HRS recommendations for the standardization and interpretation of the electrocardiogram: Part V: Electrocardiogram changes associated with cardiac chamber hypertrophy: A scientific statement from the american heart association electrocardiography,” Circulation, vol. 119, no. 10, pp. e251–e261, 2009. 19228820 10.1161/CIRCULATIONAHA.108.191097

[3] L. Bacharova and E. H. Estes, “Left ventricular hypertrophy by the surface ECG,” J. Electrocardiol., vol. 50, no. 6, pp. 906–908, Nov. 2017. 28651797 10.1016/j.jelectrocard.2017.06.006

[4] B. P. Hsieh, M. X. Pham, and V. F. Froelicher, “Prognostic value of electrocardiographic criteria for left ventricular hypertrophy,” Amer. Heart J., vol. 150, no. 1, pp. 161–167, 2005. 16084164 10.1016/j.ahj.2004.08.041

[5] S. W. Rabkin, “Searching for the best machine learning algorithm for the detection of left ventricular hypertrophy from the ECG: A review,” Bioengineering, vol. 11, no. 489, 2024, Art. no. 489. 10.3390/bioengineering11050489 PMC11117908 38790356

[6] F. D. l. G. Salazar, M. E. R. Ibarguengoitia, J. R. A. López, and A. G. Cantú, “Optimizing ECG to detect echocardiographic left ventricular hypertrophy with computerbased ECG data and machine learning,” PLoS One, vol. 16, no. 11, pp. 1–14, 2021. 10.1371/journal.pone.0260661 PMC8631676 34847202

[7] R. Sparapani , “Detection of left ventricular hypertrophy using Bayesian additive regression trees: The MESA,” J. Amer. Heart Assoc., vol. 8, no. 5, 2019, Art. no. e009959. 10.1161/JAHA.118.009959 PMC6474924 30827132

[8] R. Jothiramalingam, A. Jude, R. Patan, M. Ramachandran, J. H. Duraisamy, and A. H. Gandomi, “Machine learning-based left ventricular hypertrophy detection using multi-lead ECG signal,” Neural Comput. Appl., vol. 33, no. 9, pp. 4445–4455, 2021.

[9] E. Angelaki , “Detection of abnormal left ventricular geometry in patients without cardiovascular disease through machine learning: An ECG-based approach,” J. Clin. Hypertension, vol. 23, no. 5 pp. 935–945, 2021. 10.1111/jch.14200 PMC8678829 33507615

[10] D. Y. Lim , “Machine learning versus classical electrocardiographic criteria for echocardiographic left ventricular hypertrophy in a pre-participation cohort,” Kardiologia Polska, vol. 79, no. 6, pp. 654–661, 2021. 33885269 10.33963/KP.15955

[11] C. W. Liu , “Left ventricular hypertrophy detection using electrocardiographic signal,” Sci. Rep., vol. 13, pp. 1–13, 2023. [Online]. Available: https://doi.org/10.1038/s41598-023-28325-5 36781924 10.1038/s41598-023-28325-5 PMC9924839

[12] J. M. Kwon , “Comparing the performance of artificial intelligence and conventional diagnosis criteria for detecting left ventricular hypertrophy using electrocardiography,” Europace, vol. 22, no. 3, pp. 412–419, 2020. 31800031 10.1093/europace/euz324

[13] P. Wagner , “PTB-XL, a large publicly available electrocardiography dataset,” Sci. Data, vol. 7, no. 1, pp. 1–15, Dec. 2020. 32451379 10.1038/s41597-020-0495-6 PMC7248071

[14] M. A. Reyna , “Classification of 12-lead ECGs: The physioNet/computing in cardiology challenge 2020,” Physiol. Meas., vol. 41, no. 12, 2020, Art. no. 124003. 10.1088/1361-6579/abc960 PMC8015789 33176294

[15] N. Pilia, C. Nagel, G. Lenis, S. Becker, O. Dössel, and A. Loewe, “ECGdeli - An open source ECG delineation toolbox for MATLAB,” SoftwareX, vol. 13, 2021, Art. no. 100639.

[16] C. Bock, P. Kovacs, P. Laguna, J. Meier, and M. Huemer, “ECG beat representation and delineation by means of variable projection,” IEEE Trans. Biomed. Eng., vol. 68, no. 10, pp. 2997–3008, Oct. 2021. 33571084 10.1109/TBME.2021.3058781

[17] R. Sassi and L. T. Mainardi, “An estimate of the dispersion of repolarization times based on a biophysical model of the ECG,” IEEE Trans. Biomed. Eng., vol. 58, no. 12, pp. 3396–3405, Dec. 2011. 21878404 10.1109/TBME.2011.2166263

[18] P. Laguna, J. P. Martinez Cortes, and E. Pueyo, “Techniques for ventricular repolarization instability assessment from the ECG,” Proc. IEEE, vol. 104, no. 2, pp. 392–415, Feb. 2016.

[19] A. Van Oosterom, “The dominant T wave and its significance,” J. Cardiovasc. Electrophysiol., vol. 14, no. 10, pp. 180–187, 2003. 10.1046/j.1540.8167.90309.x 14760922

[20] L. Breiman, “Random forests LEO,” Mach. Learn., vol. 45, pp. 5–32, 2001.

[21] C. C. Chang and C. J. Lin, “LIBSVM: A library for support vector machines,” ACM Trans. Intell. Syst. Technol., vol. 2, no. 3, pp. 1–40, 2001.

[22] F. E. Oguz, A. Alkan, and T. Schöler, “Emotion detection from ECG signals with different learning algorithms and automated feature engineering,” Signal, Image Video Process., vol. 17, pp. 3783–3791, 2023.

[23] F. Pedregosa , “Scikit-learn: Machine learning in python,” J. Mach. Learn. Res., vol. 12, no. 9, pp. 2825–2830, 2011.

[24] F. J. Ferri, P. Pudil, M. Hatef, and J. Kittler, “Comparative study of techniques for large-scale feature selection,” Mach. Intell. Pattern Recognit., vol. 16, pp. 404–413, 1994.

[25] S. M. Lundberg and S.-i. Lee, “A unified approach to interpreting model predictions,” 2017, *arXiv:1705.07874*.

[26] T. Lewis, “Observations upon ventricular hypertrophy with special reference to preponderance of one or the other chamber,” Heart, vol. 5, pp. 367–402, 1914.

[27] R. Gubner and H. E. Ungerleider, “Electrocardiographic criteria of left ventricular hypertrophy: Factors determining the evolution of the electrocardiographic patterns in hypertrophy and bundle branch block,” Arch. Intern. Med., vol. 72, no. 2, pp. 196–209, 1943.

[28] M. Sokolow and T. P. Lyon, “The ventricular complex in left ventricular hypertrophy as obtained by unipolar precordial and limb leads,” Amer. J. Med., vol. 37, no. 2, pp. 161–186, 1949. 10.1016/0002-8703(49)90562-1 18107386

[29] E. Goldberger, Unipolar Lead Electrocardiography: Including Standard Leads, Augmented Unipolar Extremity Leads and Multiple Unipolar Precordial Leads, and a Section on Cardiac Arrhythmias. 2nd ed. Philadelphia, PA, USA: Lea & Febiger, 1949.

[30] J. Schack, R. Rosenman, and L. Katz, “The aV limb leads in the diagnosis of ventricular strain,” Amer. Heart J., vol. 40, no. 5, pp. 696–705, 1950. 14783065 10.1016/0002-8703(50)90200-6

[31] D. W. Romhilt and E. H. Estes, “A point-score system for the ECG diagnosis of left ventricular hypertrophy,” Amer. Heart J., vol. 75, no. 6, pp. 752–758, 1968. 4231231 10.1016/0002-8703(68)90035-5

[32] F. N. Wilson , “The precordial electrocardiogram,” Amer. Heart J., vol. 27, no. 1, pp. 19–85, 1944.

[33] A. Mazzoleni, R. Wolff, L. Wolff, and L. Reiner, “Correlation between component cardiac weights and electrocardiographic patterns in 185 cases,” Circulation, vol. 30, no. 6, pp. 808–829, 1964. 14246325 10.1161/01.cir.30.6.808

[34] D. W. Romhilt , “A critical appraisal of the electrocardiographic criteria for the diagnosis of left ventricular hypertrophy,” Circulation, vol. 40, no. 2, pp. 185–195, 1969. 4240354 10.1161/01.cir.40.2.185

[35] M. L. Murphy , “Reevaluation of electrocardiographic criteria for left, right and combined cardiac ventricular hypertrophy,” Amer. J. Cardiol., vol. 53, no. 8, pp. 1140–1147, 1984. 6230928 10.1016/0002-9149(84)90651-9

[36] R. Grant, Clinical Electrocardiography: The Spatial Vector Approach. New York, NY, USA: McGraw-Hill Blakiston Division, 1957.

[37] D. Holt and D. Spodick, “The Rv6:Rv5 voltage ratio in left ventricular hypertrophy,” Amer. Heart J., vol. 63, no. 5, 1962, Art. no. 339. 10.1016/0002-8703(62)90221-1 13908563

[38] J. McPhie, “Left ventricular hypertrophy: Electrocardiographic diagnosis,” in Australas. Ann. Med., vol. 7, no. 4, pp. 317–327, 1958. 13607332 10.1111/imj.1958.7.4.317

[39] L. Wolff, Electrocardiography: Fundamentals and Clinical Application, 2nd ed. Philadelphia, PA, USA: WB Saunders, 1956.

[40] R. J. Siegel and W. C. Roberts, “Electrocardiographic observations in severe aortic valve stenosis: Correlative necropsy study to clinical, hemodynamic, and ECG variables demonstrating relation of 12-lead QRS amplitude to peak systolic transaortic pressure gradient,” Amer. Heart J., vol. 103, no. 2, pp. 210–221, 1982. 6459734 10.1016/0002-8703(82)90494-x

[41] T. J. Molloy, M. Peter, R. B. Devereux, and P. Kligfield, “Electrocardiographic detection of left ventricular hypertrophy by the simple QRS voltage-duration product,” J. Amer. College Cardiol., vol. 20, no. 5, pp. 1180–1186, 1992. 10.1016/0735-1097(92)90376-x 1401620

