# Supplementary material for: An ECG-Based Model for Left Ventricular Hypertrophy Detection: A Machine Learning Approach
Source: IEEE Open J Eng Med Biol. 2024 Nov 29;6:219–26. doi: 10.1109/OJEMB.2024.3509379 (PMC11655100; doi:10.1109/OJEMB.2024.3509379)
Supplement: Supplementary Materials [file supp1-3509379.pdf]

Supplementary Materials

An ECG-based model for left ventricular hypertrophy detection: a machine learning approach

Marion Taconné, Valentina D.A. Corino, and Luca Mainardi, *Member, IEEE*

THIS Supplementary Materials provides the figures not proposed in the manuscript. Two other classifiers were also tested, and their results are provided here.

First, figure 1 represents the first four Hermite functions (Φ_i) used in this study. Indeed, thanks to its shapes, which well mimic QRS waveforms, few Hermite functions are needed to describe the QRS.

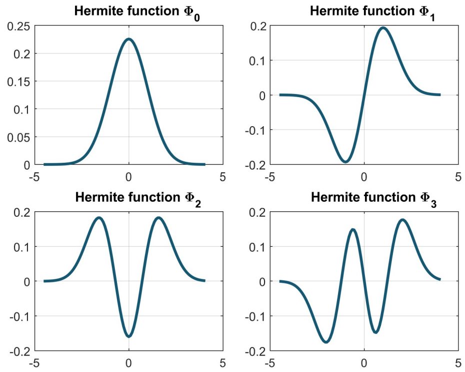

Fig. 1. The first four Hermite functions (Φ_0, Φ_1, Φ_2 and Φ_3).

Then, Figure 2 presents two examples of Hermite approximations for two QRS complexes, each with different RMSE values.

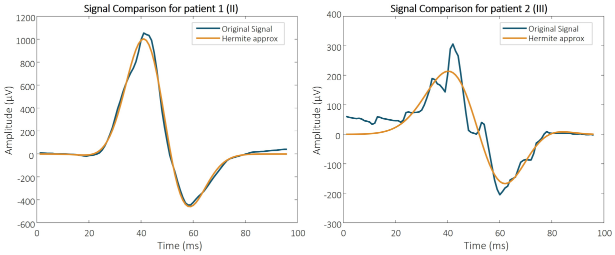

Fig. 2. Two examples of Hermite approximation for two LVH patients on different leads (II and III).

It can be observed that Hermite functions better fit signals without any fragmentation in the QRS complex. The simpler the QRS morphology is, the lower is the fitting error. Additionally, the quality of the cropped signal could impact the Hermite approximation.

Figure 3 illustrates the importance of each feature in the classification decision. It presents the results of the SHAP framework applied to the three classifiers constructed with a maximum of 30 features. One notable aspect of these results is the ability to understand the impact of each feature on the validation database and how it influences the classification outcome.

Similarly to the logistic regression , random forest, and support vector machine, the K-Nearest Neighbors and AdaBoost classifiers were also tested. Their results are presented in the same format as the previous classifiers with the ordered list of selected features (Table I) and the evaluation metrics on the validation database (Table II). The same evaluation metrics were used and are detailed here: sensitivity, specificity, accuracy, and balanced accuracy:

$$sensitivity = \frac{TP}{TP+FN} \tag{1}$$

$$specificity = \frac{TN}{TN+FP} \tag{2}$$

$$accuracy = \frac{TP+TN}{TP+TN+FP+FN} \tag{3}$$

$$balanced\ accuracy = \frac{sensitivity+specificity}{2} \tag{4}$$

where TP, TN, FP and FN are true positive, true negative, false positive and false negative respectively. The same python library: Scikit-Learn [1] were used as well. The K of the K-nearest neighbors was set at 5 and the number of estimator in the Adaboost classifier is 10.

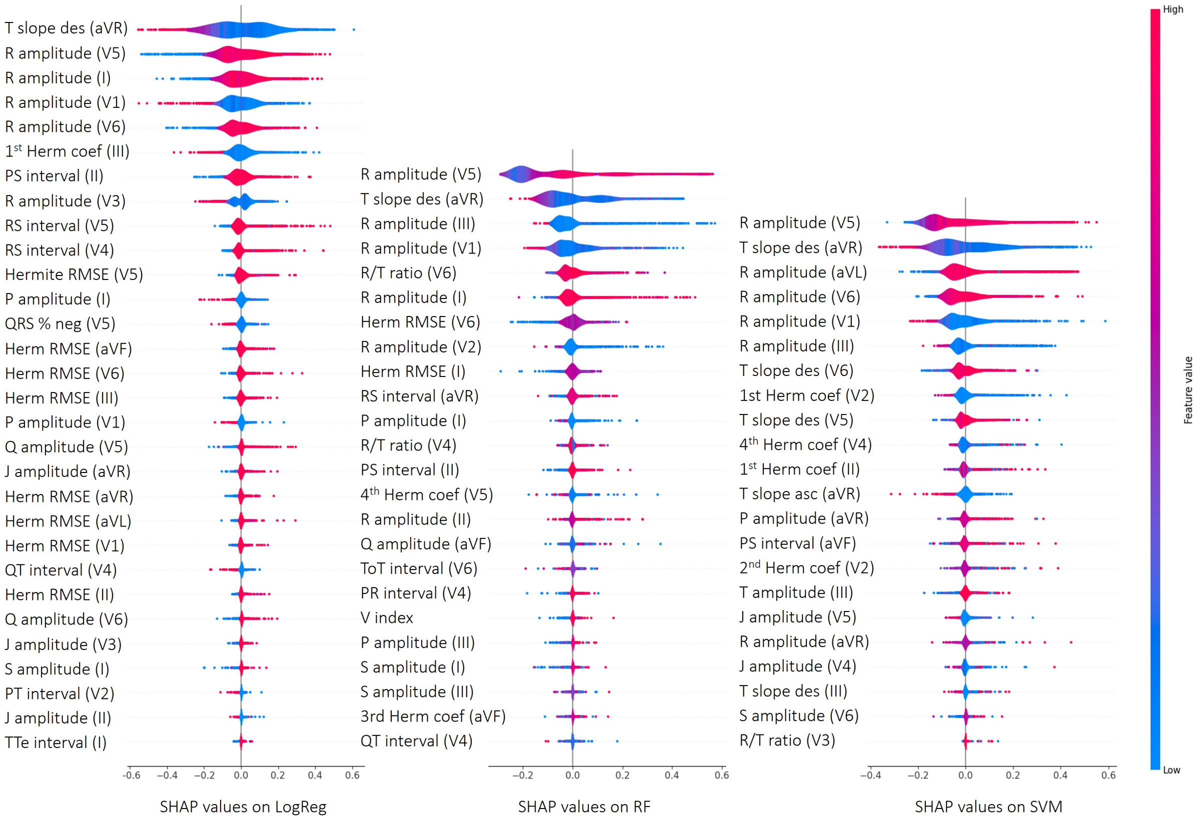

Fig. 3. SHAP values of the three algorithms: Logistic regression (LogReg), random forest (RF), and support vector machine (SVM) with up to 30 features, trained on the PTB-XL database.

N	KNN	Ada
1	R/T (aVR)	R/T (I)
2	1st Herm coef (I)	R amplitude (V5)
3	R/T (V5)	R amplitude (aVL)
4	R/P (aVF)	T amplitude (aVR)
5	R/P (V3)	R amplitude (V2)
6	R/T (V1)	RS interval (V5)
7	1st Herm coef (aVL)	T slope des (V5)
8	2nd Herm coef (aVL)	T slope des (aVR)
9	3rd Herm coef (aVL)	R amplitude (III)
10	4th Herm coef (aVL)	R amplitide (V6)
11	3rd Herm coef (V3)	
12	4th Herm coef (V3)	
13	T amplitude (aVR)	
14	R amplitude (V5)	
15	R/T (V6)	
16	1st Herm coef (V3)	
17	R amplitude (aVL)	
18	R amplitude (V1)	
19	Herm RMSE (V5)	
20	T slope des (V6)	
21	T slope asc (V6)	
22	1st Herm coef (V6)	
23	R/T (V4)	
24	R/P (I)	
25	R/T (I)	
26	R/P (aVR)	
27	R/P (V1)	
28	Herm RMSE (I)	
29		
30		

TABLE I. Features selected by the SFFS for the models: KNearest Neighbors (KNN), AdaBoost (Ada).

	Sensitivity	Specificity	Accuracy	Balanced accuracy
Ensemble KNN	0.697	0.970	0.855	0.833
Ensemble KNN5	0.445	0.927	0.770	0.741
Ensemble Ada	0.792	0.921	0.866	0.856
Ensemble Ada5	0.782	0.901	0.850	0.841

TABLE II. Sensitivity, specificity, and accuracy of the ensemble method for supplementary models on the validation database: KNearest Neighbors (KNN), AdaBoost (Ada) for the selected features up to 30 features and to the reduced to 5 features.

Studies	Population	ML method	Internal or external validation	Data and model availability
[2]	Military (2196)	SVM	Internal cross validation	Proprietary database, model not provided
[3]	Multi-ethnic (4714)	Bayesian additive regression	Internal validation	Proprietary database, model not provided
[4], [5]	Hospital (432 and 439)	C5.0 supervised ML algorithm to create a multilevel binary decision tree	External validation	Proprietary database, the simple model provided
[6]	Hospital (21,286)	ENN, LR and RF	External validation	Proprietary database, model not provided
[7]	UK biobank (32,239)	CNN	External validation	Training database accessible but not freely and external validation on proprietary database, model not provided
[8]	General population (1407)	LR, XGBoost, Random Forest, AdaBoost, Support Vector Machines	Internal cross validation	Proprietary database, model not provided
[9]	NA (528)	reservoir and ensemble ML	Internal cross validation	Proprietary database, model not provided
[10]	Military (17, 310	Random Forest, Logistic Regression, GLMNet, Random Forests, Gradient Boosting Machines	Internal cross validation	Proprietary database, model not provided
[11]	Hospital (3120)	CNN	Internal cross validation	Proprietary database, model not provided
[12]	Hospital (2456)	XGBoost	No validation, only a train and test sets	Proprietary database, model not provided
[13]	Hospital (12,008)	ENN	Internal validation	Proprietary database, model not provided
[14]	UK biobank (37,534)	LR, SVM, RF	Internal cross validation and validation tests with the different centers	Database accessible but not freely, model not provided
[15]	Military (952)	Decision tree SVM and Back propagated Neural Network	Internal cross validation	Proprietary database, model not provided

TABLE III. Summary of the LVH detection studies, based on the paper review [16]

REFERENCES

[1] F. Pedregosa, G. Varoquaux, A. Gramfort, V. Michel, B. Thirion, O. Grisel, M. Blondel, P. Prettenhofer, R. Weiss, V. Dibourg, J. Vanderplas, A. Passos, D. Cournapeau, M. Brucher, M. Perrot, and E. Duchesnay, "Scikit-learn: Machine Learning in Python," *Journal of Machine Learning Research*, vol. 12, no. 9, pp. 2825–2830, 2011.

[2] G. M. Lin and K. Liu, "An Electrocardiographic System with Anthropometrics via Machine Learning to Screen Left Ventricular Hypertrophy among Young Adults," *IEEE Journal of Translational Engineering in Health and Medicine*, vol. 8, no. December 2019, 2020.

[3] R. Sparapani, N. M. Dabbouseh, D. Gutterman, J. Zhang, H. Chen, D. A. Bluemke, J. A. Lima, G. L. Burke, and E. Z. Soliman, "Detection of Left Ventricular Hypertrophy Using Bayesian Additive Regression Trees: The MESA," *Journal of the American Heart Association*, vol. 8, no. 5, 2019.

[4] F. de la Garza-Salazar, M. E. Romero-Ibarguengoitia, E. A. Rodriguez-Diaz, J. R. Azpiri-Lopez, and A. González-Cantu, "Improvement of electrocardiographic diagnostic accuracy of left ventricular hypertrophy using a Machine Learning approach," *PLoS ONE*, vol. 15, no. 5, pp. 1–14, 2020.

[5] F. De la Garza Salazar, M. E. R. Ibarguengoitia, J. R. A. López, and A. G. Cantú, "Optimizing ECG to detect echocardiographic left ventricular hypertrophy with computerbased ECG data and machine learning," *PLoS ONE*, vol. 16, no. 11, pp. 1–14, 2021.

[6] J. M. Kwon, K. H. Jeon, H. M. Kim, M. J. Kim, S. M. Lim, K. H. Kim, P. S. Song, J. Park, R. K. Choi, and B. H. Oh, "Comparing the performance of artificial intelligence and conventional diagnosis criteria for detecting left ventricular hypertrophy using electrocardiography," *Europace*, vol. 22, no. 3, pp. 412–419, 2020.

[7] S. Khurshid, S. Friedman, J. P. Pirruccello, P. D. Achille, N. Diamant, C. D. Anderson, P. T. Ellinor, P. Batra, J. E. Ho, A. A. Philippakis, and S. A. Lubitz, "Deep Learning to Predict Cardiac Magnetic Resonance-Derived Left Ventricular Mass and Hypertrophy From 12-Lead ECGs," *Circulation: Cardiovascular Imaging*, no. June, pp. 485–495, 2021.

[8] F. Sabovčik, N. Cauwenberghs, D. Kouznetsov, F. Haddad, A. Alonso-Betanzos, C. Vens, and T. Kuznetsova, "Applying machine learning to detect early stages of cardiac remodelling and dysfunction," *European Heart Journal Cardiovascular Imaging*, vol. 22, no. 10, pp. 1208–1217, 2021.

[9] E. Angelaki, M. E. Marketou, G. D. Barmparis, A. Patrianakos, P. E. Vardas, F. Parthenakis, and G. P. Tsironis, "Detection of abnormal left ventricular geometry in patients without cardiovascular disease through machine learning : An ECG-based approach," *The Journal of Clinical Hypertension published*, no. January, pp. 935–945, 2021.

[10] D. Y. Lim, G. Sng, W. H. Ho, W. Hankun, C. H. Sia, J. S. Lee, X. Shen, B. Y. Tan, E. C. Lee, M. Dalakoti, W. K. Jie, C. K. Kwan, W. Chow, R. S. Tan, C. S. Lam, T. S. Chua, T. J. Yeo, and D. T. Chong, "Machine learning versus classical electrocardiographic criteria for echocardiographic left ventricular hypertrophy in a pre-participation cohort," *Kardiologia Polska*, vol. 79, no. 6, pp. 654–661, 2021.

[11] X. Zhao, G. Huang, L. Wu, M. Wang, X. He, J. R. Wang, B. Zhou, Y. Liu, Y. Lin, D. Liu, X. Yu, S. Liang, B. Tian, L. Liu, Y. Chen, S. Qiu, X. Xie, L. Han, and X. Qian, "Deep learning assessment of left ventricular hypertrophy based on electrocardiogram," *Frontiers in Cardiovascular Medicine*, vol. 9, 2022.

[12] A. Sammani, M. Jansen, N. M. de Vries, N. de Jonge, A. F. Baas, A. S. te Riele, F. W. Asselbergs, and M. I. Oerlemans, "Automatic Identification of Patients With Unexplained Left Ventricular Hypertrophy in Electronic Health Record Data to Improve Targeted Treatment and Family Screening," *Frontiers in Cardiovascular Medicine*, vol. 9, no. April, pp. 1–11, 2022.

[13] T. Kokubo, S. Kodera, S. Sawano, S. Katsushika, M. Nakamoto, H. Takeuchi, N. Kimura, H. Shinohara, R. Matsuoka, K. Nakanishi, T. Nakao, Y. Higashikuni, N. Takeda, K. Fujiu, M. Daimon, H. Akazawa, H. Morita, Y. Matsuyama, and I. Komuro, "Automatic Detection of Left Ventricular Dilatation and Hypertrophy from Electrocardiograms Using Deep Learning," *International Heart Journal*, vol. 63, no. 5, pp. 939–947, 2022.

[14] H. Naderi, J. Ramírez, S. Van Duijvenboden, E. R. Pujadas, N. Aung, L. Wang, C. Anwar Ahmed Chahal, K. Lekadir, S. E. Petersen, and P. B. Munroe, "Predicting left ventricular hypertrophy from the 12-lead electrocardiogram in the UK Biobank imaging study using machine learning," *European Heart Journal - Digital Health*, vol. 4, no. 4, pp. 316–324, 2023. [Online]. Available: https://doi.org/10.1093/ehjdh/ztad037

[15] C. W. Liu, F. H. Wu, Y. L. Hu, R. H. Pan, C. H. Lin, Y. Chen, G. Tseng, Y. Chan, and C. Wang, "Left ventricular hypertrophy detection using electrocardiographic signal," *Scientific Reports*, vol. 13, pp. 1–13, 2023. [Online]. Available: https://doi.org/10.1038/s41598-023-28325-5

[16] S. W. Rabkin, "Searching for the Best Machine Learning Algorithm for the Detection of Left Ventricular Hypertrophy from the ECG : A Review," *Bioengineering*, vol. 11, no. 489, 2024.
